# Non-contiguous finished genome sequence and description of *Brevibacterium senegalense* sp. nov.

**DOI:** 10.4056/sigs.3256677

**Published:** 2012-12-10

**Authors:** Sahare Kokcha, Dhamodharan Ramasamy, Jean-Christophe Lagier, Catherine Robert, Didier Raoult, Pierre-Edouard Fournier

**Affiliations:** 1 Aix-Marseille Université, Faculté de médecine,

**Keywords:** *Brevibacterium senegalense*, genome

## Abstract

*Brevibacterium senegalense* strain JC43^T^ sp. nov. is the type strain of *Brevibacterium senegalense* sp. nov., a new species within the *Brevibacterium* genus. This strain, whose genome is described here, was isolated from the fecal flora of a healthy Senegalese patient. *B. senegalense* is an aerobic rod-shaped Gram-positive bacterium. Here we describe the features of this organism, together with the complete genome sequence and annotation. The 3,425,960 bp long genome (1 chromosome but no plasmid) contains 3,064 protein-coding and 49 RNA genes.

## Introduction

*Brevibacterium senegalense* strain JC43^T^ (= CSUR P155 = DSM 25783) is the type strain of *B. senegalense*. sp. nov. This bacterium is a non-motile, rod-shaped, Gram-positive, catalase-positive bacterium that was isolated from the stool of a healthy Senegalese patient as part of a study aiming at cultivating individually all bacterial species within human feces.

Bacterial taxonomy has undergone many changes over recent years. The DNA-DNA hybridization and G+C content criteria, once considered as gold standards [1], were gradually replaced by gene sequencing. In particular, 16S rRNA sequencing has deeply changed the way bacteria and archaea are classified [2]. More recently, the development of high throughput genome sequencing methods and mass spectrometric analyses of bacteria have provided a wealth of genetic and proteomic information [3]. We recently used a polyphasic approach [4] that includes genomic data, MALDI-TOF spectrum and major phenotypic characteristics to describe new bacterial species [5-11].

The genus *Brevibacterium* (Breed 1953) [12] was created in 1953 to gather short non-spore-forming and non-branching rods. To date, this genus is comprised of Gram-positive, irregular, rod-shaped, non-acid-fast bacteria, and contains 31 recognized species with validly published names [13]. *Brevibacterium* is the type genus of the family *Brevibacteriaceae* (Breed 1953) [14]. Members of the genus *Brevibacterium* are isolated from human samples, dairy products, poultry and environmental specimens. In humans, they are found on skin surfaces [15], but have also been demonstrated to cause rare cases of bacteremia, endocarditis, pericarditis, brain abscess and peritonitis. These infections have been observed mainly in immunocompromised patients, with the exception of two cases of bacteremia in immunocompetent patients with central venous catheters [15,16]. To date, only four *Brevibacterium* species have been detected in human infection, including *B. epidermidis* (Collins *et al.* 1983) [15,17,18], *B. casei* (Collins *et al.* 1983) [16,19], *B. iodinum* (Collins *et al.* 1981) and *B. otitidis* (Pascual *et al.* 1996).

Here we present a summary classification and a set of features for *B. senegalense* sp. nov. strain JC43^T^ together with the description of the complete genomic sequencing and annotation. These characteristics support the circumscription of the *B. senegalense* species.

## Organism information

A stool sample was collected from a healthy 16-year-old male Senegalese volunteer patient living in Dielmo (rural village in the Guinean-Sudanian zone in Senegal), who was included in a research protocol. Written assent was obtained from this individual. No written consent was needed from his guardians for this study because he was older than 15 years old (in accordance with the previous project approved by the Ministry of Health of Senegal and the assembled village population, and as published elsewhere [5-11]. Both this study and the assent procedure were approved by the National Ethics Committee of Senegal (CNERS) and the Ethics Committee of the Institut Fédératif de Recherche IFR48, Faculty of Medicine, Marseille, France (agreement numbers 09-022 and 11-017)). Several other new bacterial species were isolated from this specimen using various culture conditions, including the recently described *Alistipes timonensis,* *A. senegalensis,* *Anaerococcus senegalensis,* *Bacillus timonensis*, *Clostridium senegalense*, *Paenibacillus senegalensis*, and *Peptoniphilus timonensis* [5-11]. The fecal specimen was preserved at -80°C after collection and sent to Marseille. Strain JC43^T^ (Table 1) was isolated in December 2010 after inoculation on Brucella agar (BD diagnostic, Heilderberg, Germany), in aerobic atmosphere at 37°C.

**Table 1:** Classification and general features of *Brevibacterium senegalense* strain JC43^T^ according to the MIGS recommendations [20]

**MIGS ID**	**Property**	**Term**	**Evidence code^a^**
	Current classification	Domain *Bacteria*	TAS [21]
		Phylum *Actinobacteria*	TAS [22]
		Class *Actinobacteria*	TAS [23]
		Order *Actinomycetales*	TAS [23-26]
		Family *Brevibacteriaceae*	TAS [23,24,26,27]
		Genus *Brevibacterium*	TAS [14,24]
		Species *Brevibacterium senegalensis*	IDA
		Type strain JC43^T^	IDA
	Gram stain	positive	IDA
	Cell shape	rod	IDA
	Motility	nonmotile	IDA
	Sporulation	nonsporulating	IDA
	Temperature range	Mesophile	IDA
	Optimum temperature	30 - 37°C	IDA
MIGS-6.3	Salinity	unknown	IDA
MIGS-22	Oxygen requirement	aerobic	IDA
	Carbon source	glucose	
	Energy source	chemoorganotrophic	
MIGS-6	Habitat	human gut	IDA
MIGS-15	Biotic relationship	free living	IDA
MIGS-14	Pathogenicity Biosafety level Isolation	unknown 2 human feces	
MIGS-4	Geographic location	Senegal	IDA
MIGS-5	Sample collection time	September 2010	IDA
MIGS-4.1	Latitude – Longitude	13.7167 -16.4167	IDA
MIGS-4.3	Depth	surface	IDA
MIGS-4.4	Altitude	51 m above sea level	IDA

^a^Evidence codes - IDA: Inferred from Direct Assay (first time in publication); TAS: Traceable Author Statement (a direct report exists in the literature); NAS: Non-traceable Author Statement (not directly observed for the living, isolated sample, but based on a generally accepted property of the species, or anecdotal evidence). These evidence codes are from the Gene Ontology project [28].

**Figure 1:**
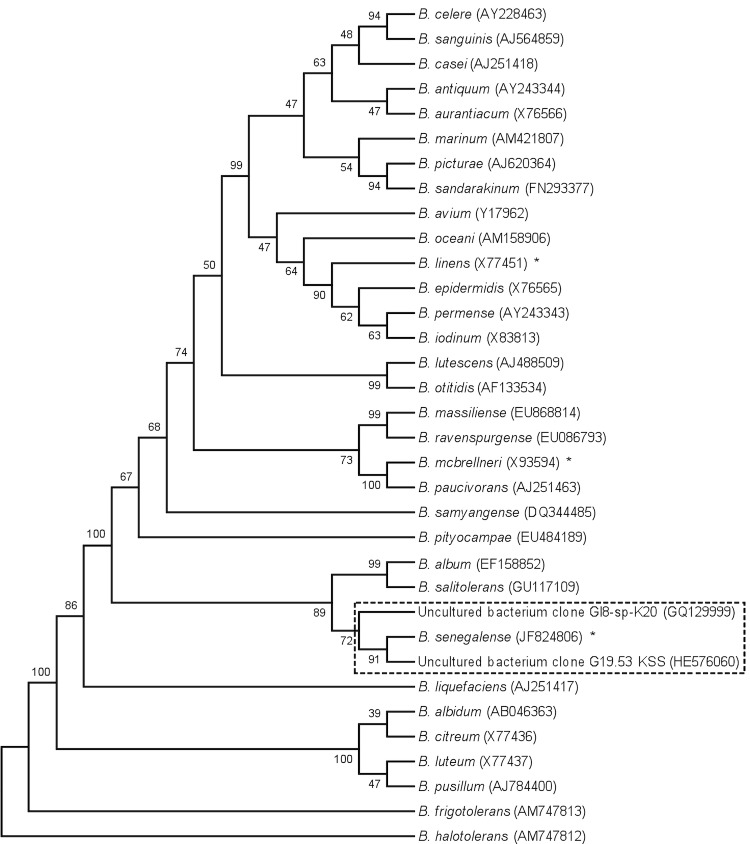
Phylogenetic tree highlighting the position of *Brevibacterium senegalense* strain JC43^T^ relative to other type strains within the genus *Brevibacterium*. GenBank accession numbers are indicated in parentheses. Sequences were aligned using CLUSTALW, and phylogenetic inferences obtained using the maximum-likelihood method within the MEGA software. Numbers at the nodes are percentages of bootstrap values obtained by repeating 500 times the analysis to generate a majority consensus tree. The dashed-line square shows sequences that exhibit degrees of similarity > 99% with *B. senegalense* (same species). Asterisks indicate the species for which genome sequences are currently available.

The strain exhibited 97.1 and 96.7% nucleotide sequence similarities with *B. salitolerans* (Guan *et al.* 2010) and *B. album* (Tang *et al.* 2008), respectively, the phylogenetically closest validated *Brevibacterium* species (Figure 1). These values were lower than the 98.7% 16S rRNA gene sequence threshold recommended by Stackebrandt and Ebers to delineate a new species without carrying out DNA-DNA hybridization [2]. In comparison to 16S sequences in the GenBank database [29], strain JC43^T^ also exhibited nucleotide sequence similarities greater than 98.7% with uncultured bacterial clones detected in water-miscible metalworking fluids [30] and on clean room surfaces [31]. These bacteria most likely belong to the same species as strains JC43 (Figure 1).

Different growth temperatures (25, 30, 37, 45°C) were tested; no growth occurred at 45°C, weak growth occurred at 25°C, and optimal growth was observed between 30 to 37°C.

Colonies were translucent and smooth, with a diameter of 1 mm on blood-enriched Columbia agar and Brain Heart Infusion (BHI) agar. Growth of the strain was tested under anaerobic and microaerophilic conditions using GENbag anaer and GENbag microaer systems, respectively (BioMérieux), and in the presence of air, of 5% CO_2_ and in aerobic conditions. Optimal growth was obtained aerobically and with 5% CO_2._ Weak growth was observed under microaerophilic conditions. No growth was observed in an anaerobic atmosphere.

**Figure 2:**
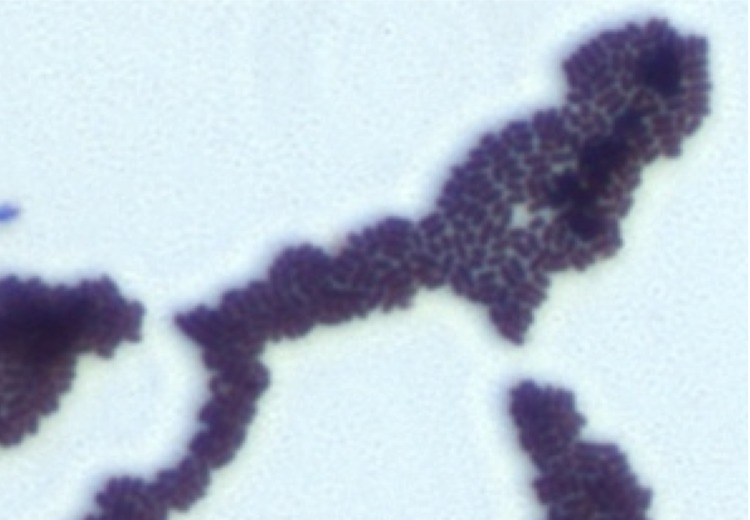
Gram staining of *B. senegalense* strain JC43^T^

**Figure 3:**
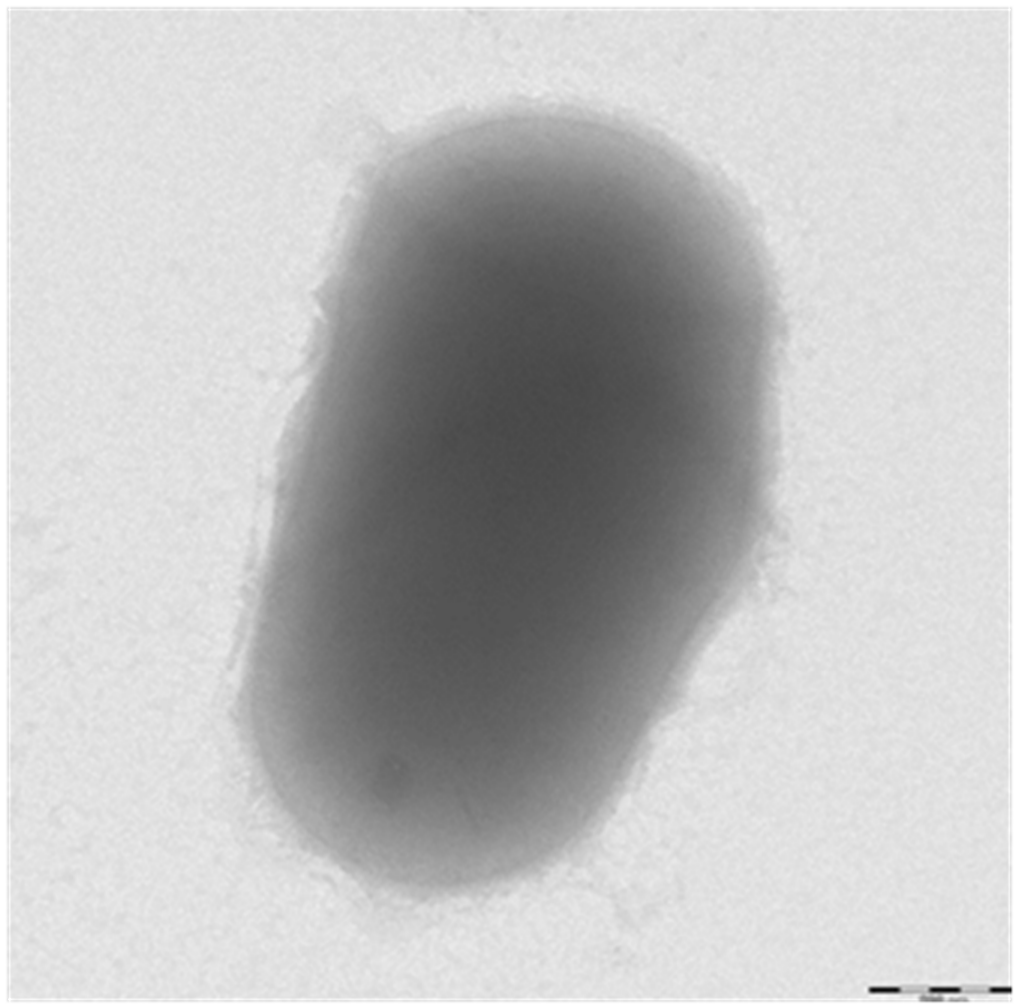
Transmission electron microscopy of *B. senegalense* strain JC43^T^, using a Morgani 268D (Philips) at an operating voltage of 60kV. The scale bar represents 200 nm.

Gram staining showed Gram-positive rods. A motility test was negative. Cells grown on agar are Gram-positive (Figure 2) and are mostly grouped in small clumps (Figure 3). Their length and width range from 0.83 to 3.86 µm (mean, 2.55 µm) and 0.57 to 0.78 µm (mean, 0.68 µm), respectively.

Strain JC43^T^ exhibited catalase activity but not oxidase activity. Using the API CORYNE system(BioMérieux), positive reactions were observed for nitrate reduction, pyrrolidonyl arylamidase, alkaline phosphatase, α-glucosidase. A weak reaction was observed for gelatin hydrolysis. Negative reactions were observed for urease, pyrazinamidase, β-glucuronidase, β-galactosidase, α-glucosidase, N-acetyl-β-glucosaminidase, β-glucosidase (aesculin hydrolysis), and acid production from D-ribose, D-glucose, D-xylose, D-mannitol, maltose, D-lactose, sucrose and glycogen. Using API ZYM (BioMérieux), positive reactions were observed for esterase (C4), esterase lipase (C8), leucine arylamidase and acid and alkaline phosphatase. Negative reactions were observed for valine arylamidase, cystine arylamidase, trypsin, α-chymotrypsin, naphtol-AS-BI-phosphohydrolase, lipase, α-galactosidase, β-galactosidase, β-glucuronidase, α-glucosidase, β-glucosidase, *N*-acetyl-β-glucosaminidase, α-mannosidase and α-fucosidase. *B. senegalense* is susceptible to penicillin G, amoxicillin, imipenem, ciprofloxacin, rifampin, gentamicin, doxycycline and vancomycin but resistant to trimethoprim/sulfamethoxazole and metronidazole. By comparison to *B. salitolerans* [32] and *B. album* [33], *B. senegalense* strain JC43^T^ differed in growth temperature, gelatin hydrolysis, pyrazinamidase, acid production from D-ribose, and nitrate reduction. In addition, *B. senegalense* also differed from the former species in β-glucosidase (aesculin hydrolysis) activity [32], and from the latter species in motility, valine arylamidase, cystine arylamidase, trypsin, α-chymotrypsin and naphtol-AS-BI-phosphohydrolase activities [33].

**Figure 4:**
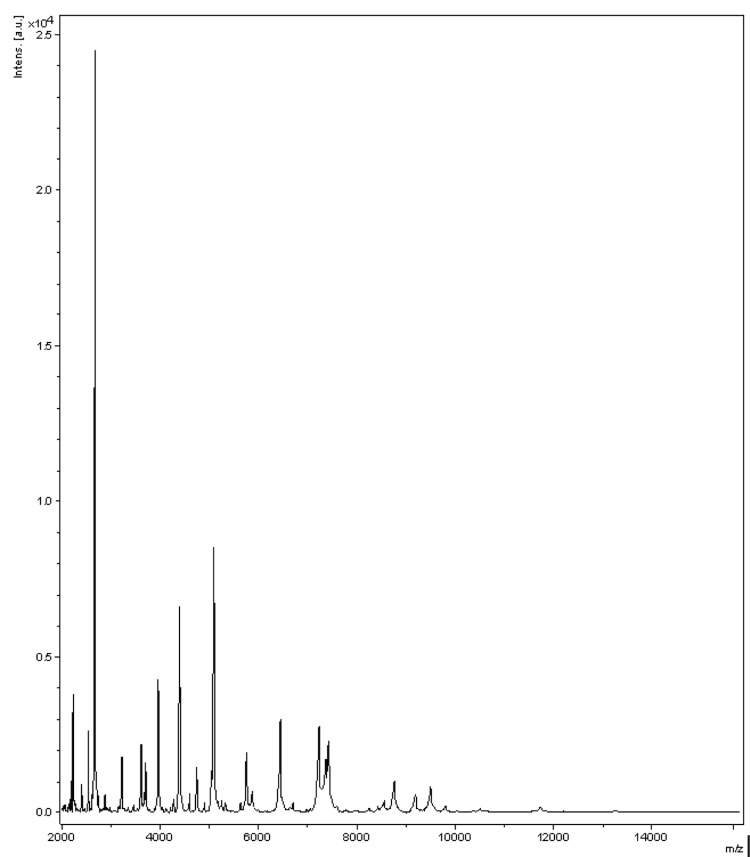
Reference mass spectrum from *B. senegalense* strain JC43^T^. Spectra from 4 individual colonies were compared and a reference spectrum was generated.

Matrix-assisted laser-desorption/ionization time-of-flight (MALDI-TOF) MS protein analysis was carried out as previously described [5,34] using a Microflex spectrometer (Bruker Daltonics, Germany). Twelve distinct deposits were done for strain JC43^T^ from four isolated colonies. The 12 JC43^T^ spectra were imported into the MALDI BioTyper software (version 2.0, Bruker) and analyzed by standard pattern matching (with default parameter settings) against the main spectra of 3,769 bacteria, which were used as reference data, in the BioTyper database. The database contained 41 spectra from 18 validly published *Brevibacterium* species, including *B. avium,* *B. celere, B. casei, B. aurantiacum, B. epidermidis, B. iodinum, B. linens, B. luteolum, B. marinum, B. massiliense, B. mcbrellneri, B. otitidis, B. paucivorans, B. picturae, B. pityocampae, B. ravenspurgense, B. sanguinis and B. stationis*. No significant score was obtained for strain JC43, thus suggesting that our isolate was not a member of a known *Brevibacterium* species within the Bruker database. We incremented our database with the spectrum from strain JC43 (Figure 4).

## Genome sequencing and annotation

### Genome project history

The organism was selected for sequencing on the basis of its phylogenetic position and 16S rRNA similarity to other members of the *Brevibacterium* genus, and is part of a study aiming at isolating all bacterial species within human feces. It was the third genome of a *Brevibacterium* species. The genome EMBL accession number is CAHK00000000 and consists of 80 contigs. Table 2 shows the project information and its association with MIGS version 2.0 compliance.

**Table 2:** Project information

**MIGS ID**	**Property**	**Term**
MIGS-31	Finishing quality	High quality draft
MIGS-28	Libraries used	One shotgun, one paired-end 3-kb
MIGS-29	Sequencing platforms	454 GS FLX Titanium
MIGS-31.2	Fold coverage	20 ×
MIGS-30	Assemblers	Newbler version 2.5.3
MIGS-32	Gene calling method	Prodigal
	EMBL ID	CAHK00000000
	EMBL date of Release	February 2, 2012
	Project relevance	Study of the human gut microbiot

### Growth conditions and DNA isolation

*B. senegalense* sp. nov. strain JC43^T^ (CSUR = P155, DSM = 25783) was grown aerobically on 5% sheep blood-enriched Columbia agar at 37°C. Seven petri dishes were spread and resuspended in 3x100µl of G2 buffer (EZ1 DNA Tissue kit, Qiagen). A first mechanical lysis was performed by glass powder on the Fastprep-24 device (Sample Preparation system; MP Biomedicals, USA) using 2x20 seconds cycles. DNA was then treated with 2.5µg/µL lysozyme (30 minutes at 37°C) and extracted through the BioRobot EZ 1 Advanced XL (Qiagen). The DNA was then concentrated and purified on a Qiamp kit (Qiagen). The yield and the concentration was measured by the Quant-it Picogreen kit (Invitrogen) on the Genios_Tecan fluorometer at 68,1 ng/µl.

### Genome sequencing and assembly

A shotgun library and a 3kb paired end library were pyrosequenced on the 454 Roche Titanium sequencing platform. This project was loaded on one 1/4 region region of PTP Picotiterplate (Roche, Meylan, France) for the shotgun library and 4 × 1/4 region for the 3-kb paired-end library. The shotgun library was constructed with 500 ng of DNA with the GS Rapid library Prep kit as described by the manufacturer (Roche). For the paired-end library, 5µg of DNA was mechanically fragmented on a Hydroshear device (Digilab, Holliston, MA, USA) with an enrichment size at 3-4kb. DNA fragmentation was visualized using an Agilent 2100 BioAnalyzer on a DNA labchip 7500 with an optimal size of 3.692 kb. The library was constructed according to the 454 Titanium paired-end protocol (Roche). Circularization and nebulization were performed and generated a pattern with an optimum of 510 bp. After PCR amplification through 15 cycles followed by double size selection, the single stranded paired-end library was then quantified using a Quant-it Ribogreen kit (Invitrogen) on a Genios Tecan fluorometer at 245 pg/µL. The library concentration equivalence was calculated at 8.80E+08 molecules/µL. The libraries were stocked at -20°C until further use.

The shotgun library was clonally amplified with 3 cpb in 3 emPCR reactions and the 3-kb paired-end library was amplified with 1 cpb in 10 emPCR reactions and 0.25 cpb in 4 emPCR with the GS Titanium SV emPCR Kit (Lib-L) v2 (Roche). The yield of the shotgun emPCR reactions was higher than expected at 24%, but the yields of the two types of paired-end emPCR were 16.7% and 11.01%, respectively, in the range of 5 to 20% from the Roche procedure.

The libraries were loaded on the GS Titanium PicoTiterPlate PTP Kit 70×75 and sequenced with the GS FLX Titanium Sequencing Kit XLR70 (Roche). The runs were performed overnight and then analyzed on the cluster through the gsRunBrowser and Newbler Assembler (Roche). A total of 752,121 passed filter wells were obtained and generated 203.1 Mb of sequence with an average length of 265 bp. The passed filter sequences were assembled using Newbler with 90% identity and 40 bp as overlap. The final assembly identified 80 contigs (>500 bp) arranged into 16 scaffolds and generated a genome size of 3.42 Mb.

### Genome annotation

Open Reading Frames (ORFs) were predicted using Prodigal [35] with default parameters but the predicted ORFs were excluded if they were spanning a sequencing GAP region. The predicted bacterial protein sequences were searched against the GenBank database and the Clusters of Orthologous Groups (COG) database using BLASTP. The tRNAScanSE tool [36] was used to find tRNA genes, whereas ribosomal RNAs were found using RNAmmer [37]. Transmembrane domains and signal peptides were predicted using TMHMM [38] and SignalP [39], respectively. ORFans were identified if their BLASTp *E-*value was lower than 1e-03 for alignment length greater than 80 amino acids. If alignment lengths were smaller than 80 amino acids, we used an *E*-value of 1e-05. To estimate the mean level of nucleotide sequence similarity at the genome level between *B. senegalense*, *B. linens* (GenBank accession number AAGP00000000) and *B. mcbrellneri* (ADNU00000000) we compared the ORFs only using BLASTN at a query coverage of ≥ 70% and a minimum nucleotide length of 100 bp.

## Genome properties

**Figure 5:**
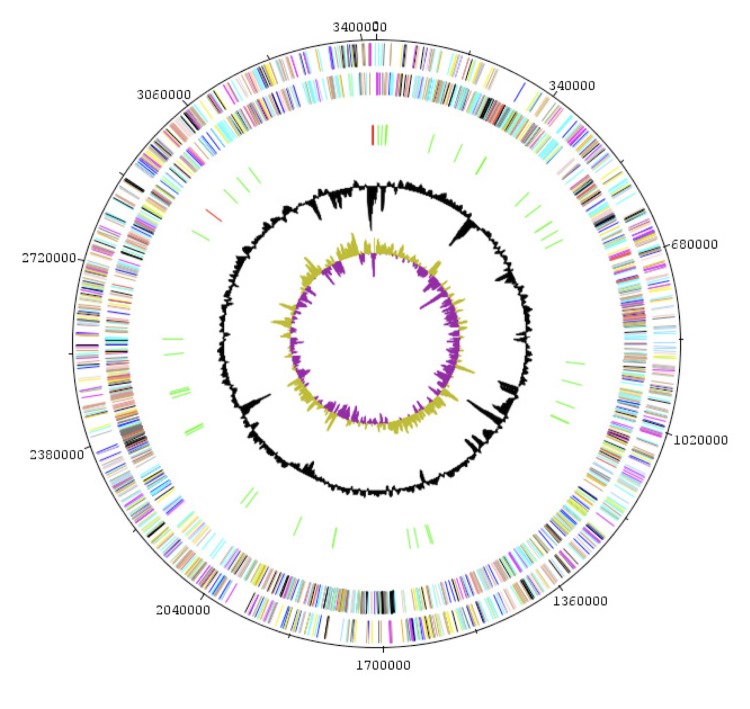
Graphical circular map of the *Brevibacterium senegalense* genome. From outside to the center: genes on the forward strand (colored by COG categories), genes on the reverse strand (colored by COG categories), RNA genes (tRNAs, green; rRNAs, red), G+C content, GC skew.

The genome is 3,425,960 bp long (1 chromosome, but no plasmid) with a 70.00% G+C content (Table 3 and Figure 5). Of the 3,114 predicted genes, 3,065 were protein-coding genes and 49 were RNAs, including 3 rRNA operons (5S, 16S and 23S rRNA) and 40 tRNAs. A total of 2,077 genes (66.7%) were assigned a putative function. The distribution of genes into COGs functional categories is presented in Table 4 and Figure 5. The properties and the statistics of the genome are summarized in Tables 3 and 4.

**Table 3:** Nucleotide content and gene count levels of the genome

**Attribute**	**Value**	**% of total^a^**
Genome size (bp)	3,425,960	100
DNA coding region (bp)	3,115,812	90.94
DNA G+C content (bp)	2,398,172	70.00
Total genes	3,114	100
RNA genes	49	1.57
Protein-coding genes	3,064	98.39
Genes with function prediction	2,378	76.36
Genes assigned to COGs	2,077	66.69
Genes with peptide signals	181	5.8
Genes with transmembrane helices	317	10.17

^a^ The total is based on either the size of the genome in base pairs or the total number of protein coding genes in the annotated genome

**Table 4:** Number of genes associated with the 25 general COG functional categories

**Code**	**Value**	**% of total**^a^	**Description**
J	149	4.86	Translation
A	1	0.032	RNA processing and modification
K	163	5.31	Transcription
L	176	5.74	Replication, recombination and repair
B	0	0	Chromatin structure and dynamics
D	24	0.78	Cell cycle control, mitosis and meiosis
Y	0	0	Nuclear structure
V	47	1.53	Defense mechanisms
T	52	1.69	Signal transduction mechanisms
M	105	3.42	Cell wall/membrane biogenesis
N	0	0	Cell motility
Z	0	0	Cytoskeleton
W	0	0	Extracellular structures
U	26	0.84	Intracellular trafficking and secretion
O	76	2.47	Posttranslational modification, protein turnover, chaperones
C	139	4.53	Energy production and conversion
G	88	2.87	Carbohydrate transport and metabolism
E	189	6.16	Amino acid transport and metabolism
F	63	2.05	Nucleotide transport and metabolism
H	72	2.34	Coenzyme transport and metabolism
I	123	4.01	Lipid transport and metabolism
P	127	4.14	Inorganic ion transport and metabolism
Q	25	0.81	Secondary metabolites biosynthesis, transport and catabolism
R	250	8.15	General function prediction only
S	182	5.93	Function unknown
-	301	9.82	Not in COGs

^a^ The total is based on the total number of protein coding genes in the annotated genome

## Genomic comparison with *B. linens* and *B. mcbrellneri*

Currently, two draft genomes from *Brevibacterium* species are available. By comparison with *B. linens* strain BL2 (GenBank accession number AAGP00000000) and *B. mcbrellneri* strain ATCC 49030 (ADNU00000000) *B. senegalense* strain JC43^T^ has a smaller genome than the former (3.42 Mb *vs* 4.37Mb) but larger than the latter (2.56Mb). *B. senegalense* also has a higher G+C content than the other two genomes (70.00% *vs* 62.8% and 58.00%, respectively); it has a smaller number of predicted genes (3,114) than *B. linens* (4,054) but greater than *B. mcbrellneri* (2,437). Finally, at the genome level, *B. senegalense* exhibited percentages of nucleotide sequence similarity of 86.28% (range 70.01-100%) and 70.19% (range 86.09-100%) with *B. linens* and *B. mcbrellneri*, respectively.

## Conclusion

On the basis of phenotypic (Table 5), phylogenetic and genomic analyses, we formally propose the creation of *Brevibacterium senegalense* sp. nov. that contains the strain JC43^T^. This bacterium originated from Senegal.

**Table 5:** Phenotypic differences observed between *B. senegalense* strain JC43T, *B. salitolerans* strain YIM90718 and *B. album* strain TRM415*.*

	*B. senegalense JC43^T^*	*B. album TRM415*	*B. salitolerans* YIM90718
Motility	-	+	-
Catalase	+	+	+
Oxydase	-	-	-
Spore-forming	-	-	-
T° of growth	25-37°C	28-45°C	15-50°C
Gelatin hydrolysis	W	+	+
Alkaline phosphatase	+	+	+
Esterase lipase C8	+	+	NA
Pyrazinamidase	-	+	+
Nitrate reduction	+	-	-
β -glucuronidase	-	-	-
β - galactosidase	-	-	-
N-acetyl- β -glucosamidase	-	-	-
β -glucosidase (aesculin hydrolysis)	-	-	+
α-glucosidase	-	-	-
urease	-	-	-
Acid production for			
D-ribose	-	+	+
D-glucose	-	-	-

### Description of *Brevibacterium senegalense* sp. nov.

*Brevibacterium senegalense* (se.ne.gal.e’n.se L. gen. neutr. n. *senegalense*, pertaining to, or originating from Senegal, the country from which the specimen that enabled isolation of *B. senegalense* was isolated.)

Colonies are translucent, smooth and have a diameter of 1 mm on blood-enriched Columbia agar and Brain Heart Infusion (BHI) agar. Cells are rod-shaped and occur mostly in small clumps. Their length and width range from 0.83 to 3.86 µm (mean, 2.55 µm) and 0.57 to 0.78 µm (mean, 0.68 µm), respectively. Optimal growth is achieved aerobically with or without CO_2._ Weak growth is observed under microaerophilic conditions. No growth is observed under anaerobic conditions. Growth occurs between 30-37°C. Cells stain Gram-positive, are non-endospore-forming, and non-motile. Catalase, nitrate reduction, pyrrolidonyl arylamidase, alkaline phosphatase, α-glucosidase, gelatin hydrolysis, esterase (C4), esterase lipase (C8), leucine arylamidase and acid and alkaline phosphatase activities are present. Urease, pyrazinamidase, β-glucuronidase, β-galactosidase, α-glucosidase, N-acetyl-β-glucosaminidase, β-glucosidase (aesculin hydrolysis), acid production from D-ribose, D-glucose, D-xylose, D-mannitol, maltose, D-lactose, sucrose and glycogen, valine aylamidase, cystine aylamidase, trypsin, α-chymotrypsin, naphtol-AS-BI-phosphohydrolase, lipase, α-galactosidase, β-galactosidase, β-glucuronidase, α-glucosidase, β-glucosidase, *N*-acetyl-β-glucosaminidase, α-mannosidase and α-fucosidase activities are absent. Oxidase activity is absent. Cells are susceptible to penicillin G, amoxicillin, imipenem, ciprofloxacin, rifampin, gentamicin, doxycycline and vancomycin, but resistant to trimethoprim/sulfamethoxazole and metronidazole.

The G+C content of the genome is 70.00%. The 16S rRNA and genome sequences are deposited in EMBL under accession numbers JF824806 and CAHK00000000, respectively.

The type strain JC43^T^ (= CSUR P 155 = DSM 25783) was isolated from the fecal flora of a healthy patient in Senegal.
